# The Biochemical Basis of Hydroxymethylglutaryl-CoA Reductase Inhibitors as Neuroprotective Agents in Aneurysmal Subarachnoid Hemorrhage

**DOI:** 10.3390/ph3103186

**Published:** 2010-10-12

**Authors:** George Kwok Chu Wong, Wai Sang Poon

**Affiliations:** Department of Surgery, Prince of Wales Hospital, Shatin, NT, Hong Kong, China; E-Mail: wpoon@surgery.cuhk.edu.hk (W.S.P.)

**Keywords:** aneurysm, hydroxymethylglutaryl-CoA reductase inhibitors, neuroprotective agent, subarachnoid hemorrhage

## Abstract

Aneurysmal subarachnoid hemorrhage (SAH) has the highest morbidity and mortality rates of all types of stroke. Many aneurysmal SAH patients continue to suffer from significant neurological morbidity and mortality directly related to delayed cerebral ischemia. Pilot clinical studies of the use of Hydroxymethylglutaryl-CoA Reductase Inhibitors (statins) in aneurysmal SAH patients have reported a reduction in delayed cerebral ischemia and better clinical outcomes. We review the biochemical effects of statins on endothelium vascular function, glutamate-mediated neurotoxicity, inflammatory changes, and oxidative injuries, with reference to their possible neuroprotective effects in aneurysmal SAH.

## Background

### Aneurysmal subarachnoid hemorrhage and delayed cerebral ischemic deficits

Subarachnoid hemorrhage (SAH) from a ruptured intracranial aneurysm is a devastating subtype of cerebral stroke that affects all ages. Although accounting for only 3% of all strokes, SAH has a disproportional influence (25%) on loss of productive life from stroke conditions, as it affects the working population (peak occurrence at 40 to 60 years) [1,2]. Aneurysmal subarachnoid hemorrhage affects about 6–23 out of 100,000 adults annually. Despite advances in procedures to remove the offending aneurysm and the attendant risk of rebleeding, high rates of death and disability still occur, and are particularly prevalent when delayed cerebral ischemic deficits (DID) result in cerebral infarction. Up to half of all surviving patients develop focal or cognitive deficits, an important cause of which is DID [3,4]. DID typically develop between three to six days after initial hemorrhage [5], and there is potential to intervene before deterioration. The positive results of a phase III clinical trial of the use of the selective calcium antagonist nimodipine for neuroprotection in SAH patients is the only current example in this area of stroke treatment [6]. However, the effect was only modest, and the search for other neuroprotective agents remains an important focus in this field. 

**Figure 1:**
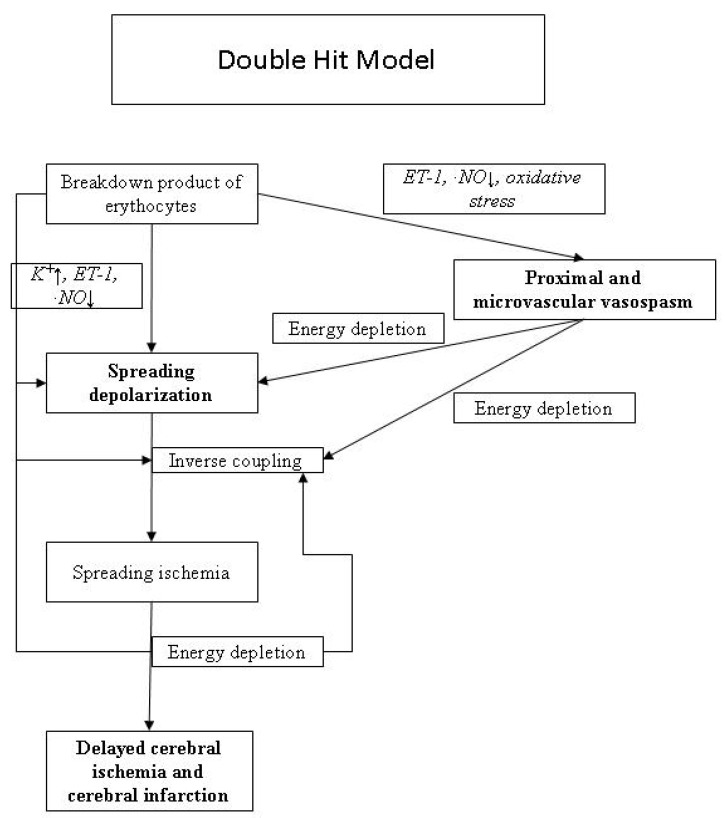
Pathophysiological mechanisms of delayed cerebral ischemia.

Cerebral vasospasm plays an important role in many cases developing DID. Ferrous hemoglobin released from subarachnoid clots leads to cerebral vasospasm by various mechanisms, including neuronal apoptosis, scavenging or decreased production of nitric oxide, increased endothelin 1 levels, direct oxidative stress in smooth muscle cells, free radical production and lipid peroxidation of cell membranes, modification of potassium and calcium channels, and differential up-regulation of genes [3]. An increase in endothelin I levels with decreased production of nitric oxide leads to raised cerebrovascular tone and vasospasm [7,8]. Oxidative stress activates myosin light chain kinase through protein kinase C and Rho kinase, leading to cerebral vessel smooth muscle wall contraction [9]. Prevention of cerebral artery vasospasm is thus the target of modern drug therapy to address the pathophysiological processes of impaired cerebral blood flow and secondary brain injury. Microcirculation vasospasm has been proposed as another important etiology for DID, resulting from increased calcium influx and suppression of voltage-dependent potassium channel current [10]. Other newly proposed contributing factors include early brain injury (blood-brain barrier disruption and delayed neuronal apoptosis) resulting from initial hemorrhage and cortical spreading depression leading to delayed infarcts [11,12]. Recently, a double hit model of DID proposed that acutely triggered microvascular spasm in response to spreading depolarization and background cerebral vasospasm were the culprit of DID (Figure 1) [3]. 

### Statins (hydroxymethylglutaryl-CoA reductase inhibitors)

**Figure 2:**
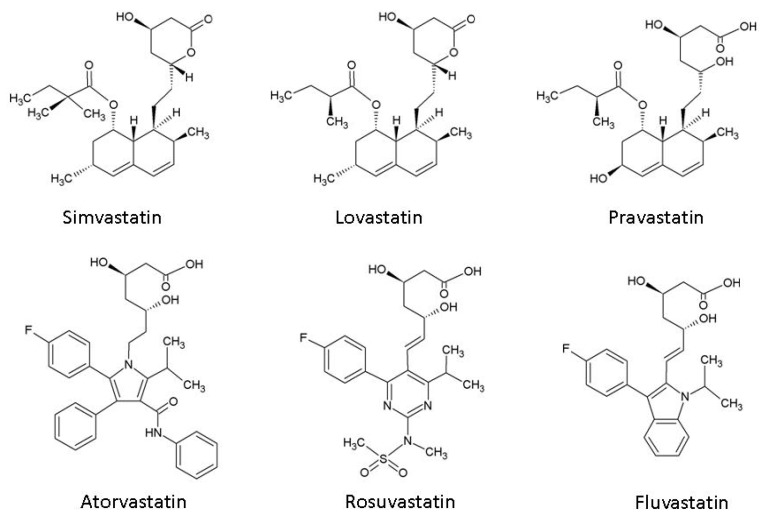
Chemical structures of 3-hydroxy-3-methylglutaryl co-enzyme A (HMG-CoA) reductase inhibitors (statins).

Statins, or 3-hydroxy-3-methylglutaryl co-enzyme A (HMG-CoA) reductase inhibitors, act by blocking the production of L-mevalonate, an intermediary product of cholesterol synthesis. Although all statins act through the same mechanism, they are divided into two categories based in their origin: the fungus-derived lovastatin, simvastatin, and pravastatin; and the synthetic atorvastatin, fluvastatin, and rosuvastatin (Figure 2) [13]. Statins are mostly taken up by the liver, but the remaining molecules bind with a high affinity to plasma protein. Statins have some side effects, such as gastrointestinal symptoms, muscle ache, hepatotoxicity (increase in serum amino transaminase levels in less than 1% of patients at a high dosage), myopathy, rash, peripheral neuropathy, insomnia, unusual dreams, and concentration problems. Myopathy, although rare, can lead to rhabdomyolysis and renal failure [13]. Almost all brain cholesterol is a product of local synthesis: 70% is associated with myelin and the rest is associated with the plasma membranes of astrocytes and neurons [14]. Brain cholesterol has an extremely long half-life. In the adult human brain, the half-life of the bulk of cholesterol is estimated to be at least five years [15]. 

**Figure 3:**
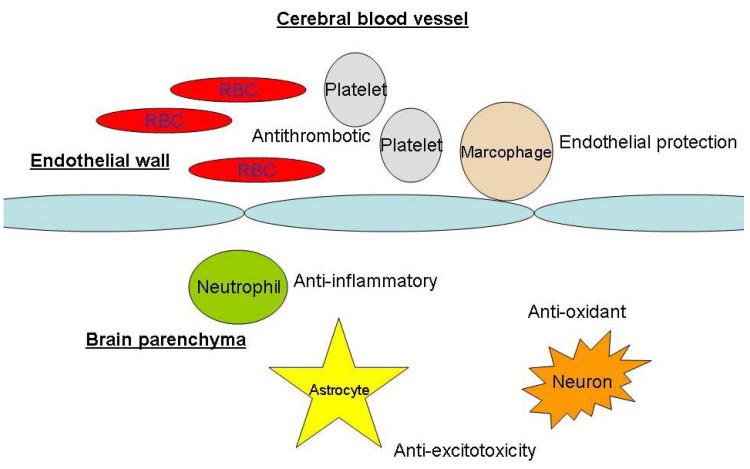
Sites of statins’ neuroprotective actions. RBC: red blood cells.

Animal experiments and human data have shown that the lipophilic statins lovastatin and simvastatin are capable of crossing the normal blood-brain barrier [16]. In addition, during the initial days after aneurysmal subarachnoid hemorrhage, experimental models had showed evidence of blood-brain barrier disruption, which could facilitate cerebral distribution of statins [17]. According to the data collected so far, there is no difference in the neuroprotective effects of lipophilic and lipophobic statins [14]. For neuroprotective effects, statins may act on the vessel wall or directly on neurons (Figure 3).

### Ongoing multi-center clinical trials of the use of simvastatin for aneurysmal subarachnoid hemorrhage

Although some phase I/II pilot studies have suggested that the prophylactic administration of statins to patients with acute aneurysmal subarachnoid hemorrhage may reduce delayed cerebral ischemia and improve clinical outcomes, current systemic reviews and meta-analyses lack the statistical power to support the routine application of statins in patients with aneurysmal subarachnoid hemorrhage [18,19]. Two ongoing multi-center and one single-center randomized controlled clinical trials have been initiated to investigate the effects of simvastatin in patients with aneurysmal subarachnoid hemorrhage.

The Simvastatin for Aneurysmal Subarachnoid Haemorrhage (STASH) is a multi-center trial led by the Cambridge Group (NCT00731627). The study is sponsored by the Cambridge University Hospitals NHS Foundation Trust and the British Heart Foundation. Based on previous positive results from phase II of the trial [20,21], the researchers hypothesize that 40 mg of simvastatin administered within 96 hours of ictus over three weeks will reduce the incidence and duration of delayed ischemic deficits following subarachnoid hemorrhage compared with a placebo, leading to improvements in both the short term (*i.e.*, a reduced need for intensive care) and the long term (six-month clinical outcome). The target sample size is 1,600 and the estimated primary completion date is June 2011.

The High-dose Simvastatin for Aneurysm Subarachnoid Haemorrhage (HDS-SAH) is a multi-center trial being conducted in Hong Kong and led by the Chinese University of Hong Kong Group (NCT01077206). The hypothesis is that a daily 80 mg (high dose) simvastatin treatment given within 96 hours of ictus over three weeks will reduce the incidence and duration of DID following subarachnoid hemorrhage compared with a daily 40mg (normal dose) simvastatin treatment, leading to improvements in clinical outcome at three months and associated advantages in terms of cost effectiveness. The target sample size is 240 and the estimated primary completion date is March 2013.

Simvastatin and Cerebral Blood Flow in Subarachnoid Hemorrhage is a single-center study run by the Washington University School of Medicine (NCT00795288). The primary objective is to investigate the effects of statin therapy on cerebral blood flow, autoregulation, cerebral oxygen extraction, and cerebral metabolism in patients with aneurysmal subarachnoid hemorrhage who are randomized to receive or not receive a daily 80 mg dose of simvastatin for 21 days in a blinded design.

## Objective of this Review

The objective of this review is to assess the current understanding of the biochemical effects of the experimental application of statins in spontaneous subarachnoid hemorrhage and whether the experimental models demonstrate that statins have a neuroprotective effect.

## Search Methodology

A PubMed Search (from inception to May 31^st^, 2010) using the keywords Hydroxymethylglutaryl-CoA Reductase Inhibitors/pharmacology*[MH] AND Neuroprotective Agents/pharmacology*[MH] yielded 55 articles. They were reviewed together with the abstracts of related citations and other references selected for their relevance to the biochemical basis of neuroprotection in aneurysmal subarachnoid hemorrhage. These articles formed the materials for the current review.

## Biochemical Basis of Neuroprotection in Aneurysmal Subarachnoid Hemorrhage and Cerebral Vasospasm

### Decrease in cerebral blood flow reduction and brain injury during cerebral artery occlusion

The ischemic effects of cerebral vasospasm can be simulated using models of cerebral artery occlusion. Endres and others concluded that chronic statin administration reduced infarct size in rats and mice after transient middle cerebral artery occlusion, with correspondingly fewer behavioral deficits [22]. The effect was confirmed with five statins and was dose dependent, indicating a class effect. The acute administration of statins also conferred similar protection in an experimental model [23]. The underlying relative increase in cerebral blood flow was probably due to the cholesterol-independent pleiotropic action of statins [24]. The most likely mechanism was an increase in the amount and activity of endothelin-derived nitric oxide synthase (eNOS) by inhibition of Rho GTPase (lengthening the half-life of eNOS mRNA) and the activation of protein kinase B, respectively [25,26,27]. eNOS converts the guanidine nitrogen of the semi-essential amino acid L-arginine to nitric oxide (NO) [28]. Vascular ·NO improves cerebral circulation by vasodilation, and this effect may be augmented by a reduction in vasoconstrictive peptide endothelin-1 [29,30] and asymmetric demethylarginine (a circulating endogenous competitive inhibitor of NO synthase) [31]. Experimental data also support the protective effect of statins against hypoxic-ischemic neuronal injury. In rat ischemic injury models, simvastatin was shown to reduce nerve fiber degeneration and attenuate endoplasmic reticulum stress response [32,33]. Statins were also shown to increase hypoxic tolerance and reduce ischemic neuronal damage in the hippocampus of mice [34,35]. In primary cortical neuronal culture, simvastatin markedly decreased oxygen and glucose deprivation/reoxygenation-evoked neuronal death [36].

### Anti-inflammatory effects

Inflammation is thought to play an important role in delayed cerebral ischemia [3]. C-reactive protein is a protein found in blood and synthesized by liver in response to inflammation and factors released by adipocytes. High sensitivity C-reactive protein (hs-CRP) was documented in large epidemiological study as an indicator of inflammation and associated with poor cardiovascular outcome, independent of other cardiovascular factors [37]. Emerging evidence showed that hs-CRP was not only a marker but also involved in the inflammatory process through CRP-induced complement activation, CRP-dependent monocyte recruitment into arterial wall, CRP-induced production of tissue factor in monocytes, CRP-blunting of endothelial vasoreactivity, CRP-induced production of cell adhesion molecules and endothelin-1, and CRP-triggered oxidation of LDL cholesterol [38]. The anti-inflammatory effects of statins were supported in a human triple crossover trial of pravastatin, simvastatin, and atorvastatin therapy [39]. Among all arms, hs-CRP concentrations were significantly reduced by 20-28%, indicating another class effect of statins. These findings concur with those of two similar studies with pravastatin and simvastatin [40,41]. The high-sensitive C-reactive protein concentration reduction was also dose dependent [42,43]. One plausible site of anti-inflammatory action was through inhibition of cellular cholesterol synthesis and increase in low density lipoprotein receptor activity in monocyte-derived macrophage in the microcirculation [44].

Statins decreased CD11b expression and the CD11B-dependent adhesion of monocytes to the endothelium and reduced the adhesiveness of monocytes isolated from patients with hypercholesterolemia [45]. Indirect evidence also suggests that statins may also reduce ischemic neuronal damage [46]. A similar effect was observed in leukocyte-endothelial cell adhesion in a rat model [47] and in neutrophil-endothelial adhesion in the coronary endothelium [48]. These effects on endothelial adhesions are postulated to protect against cerebral ischemia and reperfusion [49]. Atorvastatin reduced proinflammatory mediators such as inducible nitric oxide synthase and interleukin-4 in rat brain models [50,51]. Simvastatin also reduced the cortical induction of interleukin-1β and tumor necrosis factor-α in a rat stroke model [52]. These results indicate that statins are associated with anti-inflammatory effects in ischemic brain injury similar to that which occurs during delayed cerebral ischemia after aneurysmal subarachnoid hemorrhage.

### Anti-oxidant effects

Oxidative injury appears to be an important pathophysiology in cerebrovascular diseases [53]. The generation of free radicals causes neuronal and endothelial damage through the induction of lipid peroxidation, protein oxidation, and direct damage to nucleic acids [54]. In general, statins have been shown to reduce low-density lipoprotein oxidation [55,56]. Oxidation inhibition was dose dependent in an *in vitro* model of the hydroxyl metabolites of atrovastatin [57]. LDL oxidation by either copper ions, by the free radical system, and by macrophage-like cell line was substantially inhibited (57–97%), in a concentration-dependent manner, by pharmacological concentrations of the o-hydroxy and the p-hydroxy metabolites of atorvastatin. Similar inhibitory effects (37–96%) of the above metabolites were obtained for the susceptibility of VLDL and HDL to oxidation. Simvastatin was shown to increase α-tocopherol and result in a decrease in free-radical-mediated damage and lipid perioxidation in a 47-patient study [58] and an inhibition of low-density lipoprotein oxidation in a human monocyte-derived macrophage experimental model [59]. Statins were also shown to inhibit Rac-1-mediated NADH oxidase activity and reduce the production of reactive oxygen species in a normo-cholesterolemic, spontaneously hypertensive rat model [60].

### Anti-platelet effects

A recent clinical study suggested that delayed cerebral infarction may occur in 51% of patients after aneurysmal subarachnoid hemorrhage and may not be associated with cerebral vasospasm, which suggests a small vessel level pathology [61]. Statins are known to reduce platelet activity [62], platelet response to thrombin [63], platelet activation [64], and platelet deposition on eroded stenotic vessel walls [65]. However, whether these mechanisms are beneficial in an aneurysmal subarachnoid hemorrhage model is currently unknown. 

### Anti-excitotoxicity

Excitotoxicity caused by the overstimulation of the glutamate receptors is a major cause of neuronal death after an ischemic brain insult. In experiments using embryonic mouse neocortical cultures, treatment with statins preserved NMDA receptor-expressed cortical neurons and substantially reduced lactate dehydrogenase release caused by exposure to NMDA [66]. Neuroprotection by rosuvastatin was coincident with a decrease in cell sterols and occurred with a similar potency as inhibition of cholesterol biosynthesis. The link of cholesterol biosynthesis to anti-excitotoxicity was supported by the attenuation of neuroprotection by mevalonate or cholesterol and the similar neuroprotection achieved by the cholesterol extracting agent β-cyclodextrin. In another experiment with embryonic rat neocortical culture, atorvastatin significantly protected against glutamate-induced excitotoxicity as evidenced by propidium iodine staining, nuclear morphology, lactate dehydrogenase release, and mitochondrial tetrazolium metabolism [67]. Atorvastatin attenuated the glutamate-induced increase of intracellular calcium, which was associated with the modulation of NMDA receptor function. This mechanism may be important in counteracting the damaging effects of early brain injury and secondary insults. 

### Other neuroprotective mechanisms

Chronic poor cognitive function is common after aneurysmal subarachnoid hemorrhage [68]. In a population-based cohort study, statin consumption was associated with a reduced risk of cognitive impairment and dementia [69]. In a rat experiment after traumatic brain injury, statins increased neurogenesis, reduced neuronal death, and improved recovery in terms of spatial learning [70]. In another rat experimental model, atorvastatin ameliorated cerebral vasospasm and early brain injury after subarachnoid hemorrhage and inhibited capase-dependent apoptosis pathway [71]. It would be of interest to determine whether these protective effects can be translated to aneurysmal subarachnoid hemorrhage patients. 

### Limitations of clinical translation of experimental data

Despite the supporting experimental data for the neuroprotective effects of statins in aneurysmal subarachnoid hemorrhage, translation to clinical efficacy may not apply. The NXY-059 SAINT Trials for acute ischemic stroke patients are examples. The nitrone radical trapping agent disodium 2,4-disulfophenyl-*N*-*tert*-butylnitrone (NXY-059) has been shown to be an effective neuroprotective agent in both transient (reperfusion) and permanent focal ischemia models in rats. In both type of models, NXY-059 has a large window of opportunity, providing effective neuroprotection when given up to five hours after the start of the occlusion in transient ischemia and four hours after the start of permanent ischemia [72]. NXY-059 is also effective in marmoset permanent ischemia model in a corresponding well-tolerated plasma levels in stroke patients [73]. Although the initial SAINT I (Stroke-Acute Ischemic NXY Treatment) Trial suggested possible benefit in reducing disability when given within six hours after the onset of acute ischemic stroke [74], the subsequent SAINT II Trial and pooled analysis concluded that NXY-059 was ineffective for the treatment of acute ischemic stroke within six hours after the onset of symptoms [75,76]. NXY-59 was also ineffective on subgroup analyses based on time interval from stroke onset to onset of treatment, use or nonuse of recombinant tissue-type plasminogen activator, and NIHSS score at baseline [76]. This discrepancy is not uncommon and efficacy of new agents such as statins should be assessed by randomized controlled clinical trials with relevant clinical outcomes and proof-of-concept surrogate outcomes.

## Conclusions

Experimental data showed neuroprotective effects of statins, which might be clinically relevant to delayed cerebral ischemia and brain injury after aneurysmal subarachnoid hemorrhage. These include a decrease in cerebral blood flow reduction, anti-inflammatory effects, anti-oxidant effects, anti-platelet effects, anti-excitotoxicity, and the enhancement of recovery. The data suggest that current clinical trials of the use of statins to treat aneurysmal subarachnoid hemorrhage should continue. 
